# Comparing cardiovascular risk of patients with rheumatoid arthritis within the Social Security Disability Insurance with those commercially insured

**DOI:** 10.1186/s13075-022-02847-1

**Published:** 2022-08-22

**Authors:** Iris Navarro-Millán, Fenglong Xie, Cynthia S. Crowson, Monika M. Safford, Mangala Rajan, Sebastian E. Sattui, Jeffrey R. Curtis

**Affiliations:** 1 Weill Cornell Medicine, Division of General Internal Medicine, New York, NY USA; 2 Hospital for Special Surgery, Division of Rheumatology, New York, NY USA; 3 Division of Clinical Immunology and Rheumatology Birmingham, Faculty Office Tower 802D, University of Alabama at Birmingham, 510 20th Street South, Birmingham, AL 35294 USA; 4 Division of Clinical Trials and Biostatistics and Division of Rheumatology, Mayo Clinic, Rochester, MN USA; 5 Division of Rheumatology & Clinical Immunology, University of Pittsburgh School of Medicine, Pittsburgh, USA

**Keywords:** Rheumatoid arthritis, Cardiovascular disease, Social security disability insurance, Disability, Health outcomes

## Abstract

**Objective:**

To compare cardiovascular disease (CVD) rates in rheumatoid arthritis (RA) beneficiaries of the Social Security Disability Insurance (SSDI) with commercially insured RA patients.

**Method:**

We created three cohorts of RA patients aged < 65 years for SSDI and three for Marketscan using claims data from 2006 to 2016. The cohort definitions were as follows: (1) cohort 1: ≥ 2 diagnosis codes for RA occurring 7–365 days apart with ≥ 1 diagnosis code from a rheumatologist; (2) cohort 2: ≥ 1 diagnosis code for RA from a rheumatologist and a disease-modifying antirheumatic drugs (DMARDS); and (3) cohort 3: cohort 2, plus initiation of a new biologic/tofacitinib. We used Cox regression to determine the CVD risk comparing SSDI vs. Marketscan. Models were sequentially adjusted for age and sex (model 1); model 1 + diabetes, smoking, and high CVD risk (model 2); and model 2 + dual eligible (Medicare and Medicaid), subsidy, and state buy in (model 3).

**Results:**

There were 380,336 RA patients, mean age 53.3 (SD 8.1) years, 21–24% male. Prevalence of comorbidities was higher in SSDI vs. Marketscan. SSDI RA patients in cohort 2 (model 3) had higher CVD risk (HR 1.23 (1.14–1.33). In cohort 3 (model 3), CVD risk was not statistically significantly different between SSDI and Marketscan (HR 0.89 (0.69–1.15).

**Conclusion:**

RA patient beneficiaries of the SSDI had higher risk for CVD events than those employed. The differences in CVD events between SSDI and Marketscan were partially attributable to differences in CVD risk factors.

**Supplementary Information:**

The online version contains supplementary material available at 10.1186/s13075-022-02847-1.

## Significance and innovation

- Patients disabled with RA before retirement age 65 had 23% high risk for a cardiovascular event compared with similarly aged individuals with RA that were considered employable.
- Most of this excess risk was partially explained by differences in cardiovascular risk factors.
- Individuals disabled with RA before age 65 would likely benefit from more aggressive primary cardiovascular disease risk prevention strategies than similarly aged non-disabled individuals with RA.

## Introduction

There are 61 million adults in the USA that live with some type of disability. This translates into 26% of adults in the USA or 1 in 4 Americans [1]. The number of beneficiaries of the Social Security Disability Insurance (SSDI) has risen from 1,812,786 in 1970 to 10,153,205 in 2016 [2]. The rise has occurred despite medical advances that have allowed individuals to remain on the job and laws that ban workplace discrimination against the disabled [3]. The SSDI provides medical insurance for individuals in the USA who are disabled and younger than 65 years of age. In June 2017, beneficiaries of the SSDI accounted for roughly 15% of all Medicare beneficiaries [4]. Their cost of care was also 24% higher for these middle-aged, disabled individuals ($13,098 per capita under age 65) compared with Medicare beneficiaries over age 65 ($9972 per capita) [5]. In December 2016, diseases of the musculoskeletal system and connective tissue were the primary reason disabled workers (33%) and disabled widow(er)s (37%) received benefits [2].

Cardiovascular disease (CVD) remains the leading cause of death in patients with rheumatoid arthritis (RA) [6, 7]. Despite the risk of becoming disabled that still exists among patients with musculoskeletal conditions, such as RA, there is limited data regarding the CVD outcomes among beneficiaries of the SSDI. CVD risk estimation across diverse RA cohorts may be challenging given the potential heterogeneity in comorbidities and widely varying prevalence of CVD risk factors. The generalizability of CVD risk among middle-aged disabled patients with RA enrolled in SSDI to similarly aged patients who are commercially insured (considered to be employed) is unclear. Given the high mortality from the CVD and the high risk of disability among patients with RA, it is important to understand this risk to inform future treatment guidelines or ways to implement better existing ones. Our hypothesis was that disabled patients with RA (SSDI beneficiaries with RA younger than 65 years of age) would have higher CVD risk compared with patients wtih RA with similar age and sex who were commercially insured (Marketscan population, considered to be employable), but this difference would in part be substantially explained by multimorbidity.

## Methods

### Study design and patients

This was a retrospective longitudinal analysis from 2006 to 2016. We created 6 cohorts of patients with RA using claims data among SSDI beneficiaries and those from Marketscan [8]. Patients with RA were older than 40 years of age but younger than 65. In each dataset, *cohort 1* was defined as ≥ 2 diagnosis codes for RA occurring 7–365 days apart with ≥ 1 diagnosis code from a rheumatologist. *Cohort 2* was defined as ≥ 1 diagnosis code for RA from a rheumatologist followed by use of disease-modifying antirheumatic drugs (DMARDS, conventional synthetic (cs), biologic (b), or targeted synthetic (ts)) (Supplement Table 1). *Cohort 3* was defined as cohort 2, plus initiation of a new bDMARD or tofacitinib, used as a proxy for greater RA disease activity. Medication exposures were identified using national drug code (NDC) for oral or injection drug and Health Common Procedure Coding System (HCPCS) code for infusion drug. We excluded other autoimmune diseases (ankylosing spondylitis, inflammatory bowel disease, psoriatic arthritis, psoriasis), malignancy, human immunodeficiency virus (HIV), past myocardial infarction (MI), or stroke identified using International Classification of Diseases (ICD)-9/10-Clinical Modification (CM) diagnosis codes (Supplement Table 2) with all available data prior to follow. A patient with RA could contribute at most one episode in cohorts 1 and 2 and contribute at most one episode for each bDMARD in cohort 3.

### Exposure, comparator, and outcomes

The exposure were patients with RA who were beneficiaries of the SSDI. The comparator were patients with RA with private insurance (Marketscan claims). The outcomes were MI, defined as at least one ICD-9/10-CM (Supplement Table 2) for MI from hospital discharge with at least one night stay in hospital, or stroke, defined as at least one ICD-9/10-CM (Supplement Table 2) for stroke from hospital discharge.

### Covariates

We used all available data from the Clinical Classification Software for ICD-9-CM and ICD-10-CM developed by Agency for Healthcare Research and Quality (AHRQ) prior to the start of follow-up to define diabetes and hypertension. History of CVD risks factors were based on ICD-9/10-CM diagnosis codes using all available data. Obesity, chronic kidney disease, and hospitalized infection were defined using ICD-9/10-CM in 1-year baseline. Number of physician visits were counted as visit days in 1-year baseline. Prior bDMARDS use was identified using NDC and HCPCS code using all available data, and statin and oral prednisone use were identified using NDC code in 1-year baseline. Other covariates were dual eligibility status (Medicare and Medicaid), health insurance subsidy, and state buy-in.

### Statistical analysis

Follow-up started at the earliest date of meeting the cohort definition and 1 year of medical and pharmacy coverage and ended at earliest of (1) a CVD outcome (hospitalized MI or stroke), (2) end of enrollment, (3) age 65, and (4) end of biologic exposure plus 90 days (cohort 3 only). Descriptive statistics included standardized mean differences (SMDs) and CVD incidence rates (IR), using Poisson regression to generate 95% confidence intervals (CI). Cox regression was used to generate hazard ratio (HR), comparing enrollment in SSDI vs. Marketscan. The proportional hazard assumption was tested using numerical methods of Lin, Wei, and Ying [9]. Robust sandwich estimate of Lin and Wei was used for adjusting the effect of one RA patient’s contribution to multiple episodes in cohort 3 [10]. We selected the variables for the final models in the following way. We conducted bivariable analysis for each of the variables for each cohort. Those covariates that caused a change in the point estimates of 10% or more were considered confounders and were included in the models. Models were sequentially adjusted for age and sex (model 1), model 1 + diabetes, smoking, hypertension, high CV risk (model 2), and model 2 + a variety of additional risk factors (model 3). All analyses were conducted using SAS 9, and the University of Alabama at Birmingham, Institutional Review Board, approved this study.

## Results

There was a total of 338,792 RA patients with mean age 54.8 (SD 6.5) years of age for SSDI and 53.4 (SD 6.7) years of age for Marketscan, and 22–24% of them were males. All comorbidities and medication used were more prevalent in the SSDI population than in the Marketscan population. Patients with RA in the SSDI had a higher number of hospitalizations and physician visits than patients with RA in Marketscan (Table 1). There were 3291 MI and 2142 and stroke events in the SSDI, and there were a total of 1517 MI and 1405 stroke events in Marketscan. The mean follow-up was 3.3 years in the SSDI and 2.4 years in Marketscan.

**Table 1:** Distribution of characteristics of patients with rheumatoid arthritis by cohort and data source

	**Cohort 1**			**Cohort 2**			**Cohort 3**		
	SSDI *N* = 135,886	Marketscan *N* = 202,906	SMD	SSDI *N* = 119,516	Marketscan *N* = 186,441	SMD	SSDI *N* = 39,972	Marketscan *N* = 49,686	SMD
Age in years, mean (SD)	54.8 (6.5)	53.4 (6.7)	0.20	54.7 (6.5)	53.4 (6.7)	0.21	54.3 (6.5)	53.1 (6.6)	0.20
Age,%			0.20			0.20			0.19
40–44	8.8	12.6		8.8	12.8		9.4	13.0	
45–49	14.0	17.2		14.1	17.5		15.5	17.9	
50–54	21.3	22.6		21.4	22.6		22.2	24.0	
55–59	26.7	25.0		26.7	24.8		26.4	25.3	
60–64	29.2	22.7		29.0	22.3		26.5	19.8	
Male, %	21.9	23.9	0.05	21.3	23.6	0.06	16.9	20.9	0.10
Diabetes, %	31.9	15.4	0.40	31.5	15.1	0.40	38.2	19.5	0.42
Hypertension, %	61.6	38.5	0.48	61.0	37.8	0.48	68.8	46.1	0.47
High risk CVD, %	21.1	8.2	0.37	20.4	7.9	0.37	24.8	10.3	0.39
Obesity, %	7.0	2.7	0.20	6.9	2.7	0.20	9.2	4.6	0.18
Smoking, %	20.4	4.4	0.50	20.0	4.2	0.50	22.5	5.0	0.53
CKD, %	5.8	1.6	0.23	5.3	1.5	0.21	5.0	1.7	0.18
Glucocorticoids, %	59.6	53.5	0.12	62.3	56.4	0.12	73.4	72.5	0.03
csDMARD, %	69.8	66.0	0.08	78.7	66.0	0.29	90.5	89.9	0.02
bDMARD, %	29.6	27.7	0.04	33.9	30.6	0.07	52.5	46.5	0.12
tsDMARD, %	0.5	0.4	0.01	0.5	0.4	0.01	1.9	2.2	0.01
NSAIDS, %	56.1	55.6	0.01	56.8	57.0	0.00	57.8	58.0	0.00
Statin, %	31.2	21.8	0.21	31.5	22.3	0.21	31.7	22.1	0.22
Hospitalizations, %	22.9	9.6	0.37	21.8	9.2	0.36	22.4	10.9	0.31
Number of physician visit, mean (SD)	16.1 (10.7)	11.3 (7.2)	0.53	15.9 (10.6)	11.1 (7.2)	0.54	17.9 (11.1)	13.6 (7.8)	0.45

*SSDI* Social Security Disability Insurance

Cohort: 1 = two diagnosis code for rheumatoid arthritis (RA), at least 1 from rheumatologist; 2 = one diagnosis code for RA followed by use of disease-modifying antirheumatic drugs (DMARDS, biologic or small molecule); 3 = cohort 2 plus initiation of a biologic or tofacitinib

*CKD* chronic kidney disease, *SMD* standardized mean difference, *Other CVD* other cardiovascular disease defined as coronary artery disease including angina pectoris, peripheral artery disease or atherosclerosis, transient ischemic attack, heart failure, atrial fibrillation, aortic aneurysm or dissection, and nontraumatic intracranial hemorrhage (not leading to stroke)

**Fig. 1:**
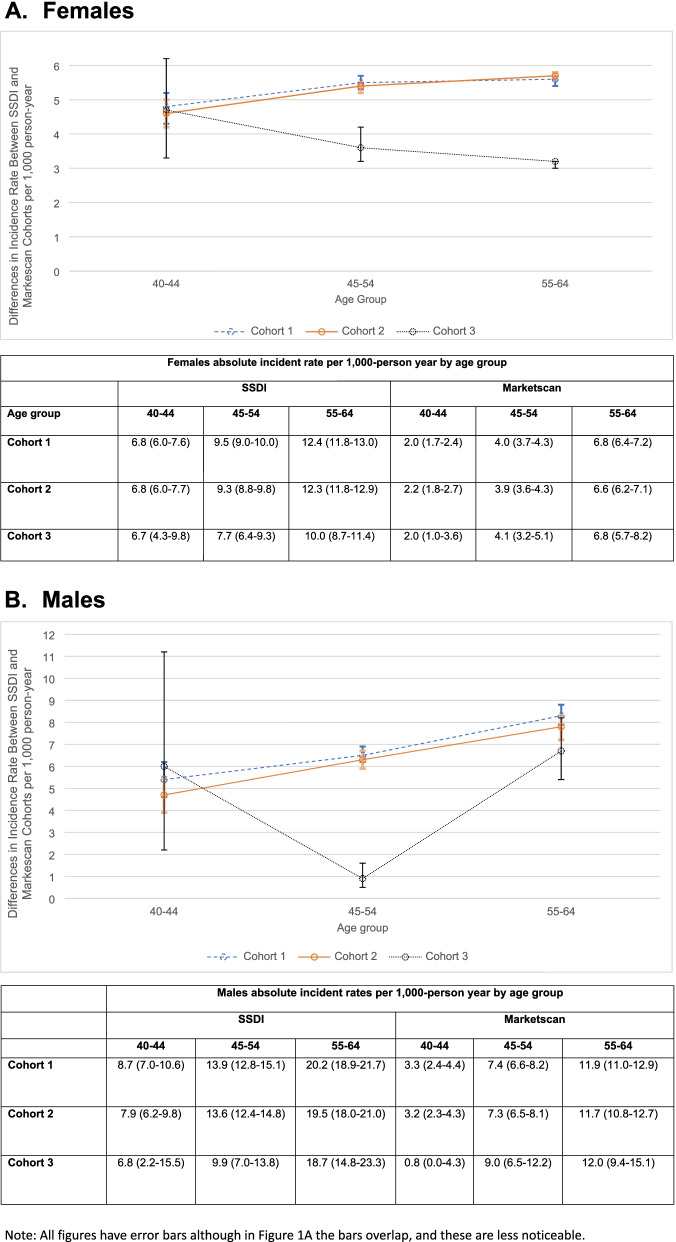
Sex- and age-specific absolute incidence rate and incidence rate differences for myocardial infarction (MI) or stroke between cohorts of patients with rheumatoid arthritis from Marketscan and beneficiaries of the Social Security Disability Insurance (SSDI)

The IR per 1000-person year for MI or stroke was higher in the SSDI than in Marketscan and higher among men. Figure 1A shows differences in MI or stroke within females with RA enrolled in the SSDI and Marketscan for ages 40–44, 45–54, and 55–65. Figure 1B shows differences in MI or Stroke within men with RA in the SSDI and Marketscan for ages 40–44, 45–54, and 55–65. A table below each figure includes the information on the absolute incidence rates for MI or stroke corresponding to each cohort, by sex and age group. The IR for CVD events between patients with RA in the SSDI and Marketscan were 1.2–2.0 times greater in SSDI vs. Marketscan (Table 2 model 1). In cohort 1, where we did not condition on drug initiation, the risk for MI was higher among SSDI beneficiaries compared with those in Marketscan with a HR of 1.46 (95% CI 1.33–1.60). In terms of absolute incidence rates, this 46% increased risk translates into an absolute difference of 1.32 more myocardial infarcts per 1000 person-years in the SSDI versus Marketscan. Similar findings were observed in cohort 2 where SSDI beneficiaries had higher risk for MI than those in Marketscan with a HR of 1.49 (95% CI 1.35–1.65). The absolute rate difference in this cohort was of 1.57 more myocardial infarct events per 1000 SSDI beneficiaries versus Marketscan.

**Table 2:** Adjusted hazard ratios and 95% CI for myocardial infarction and stroke in RA cohorts, comparing SSDI beneficiaries to Marketscan enrollees

Cohort	Outcome	Adjusted HR model 1 (95% confidence interval)^a^	Adjusted HR model 2 (95% confidence interval)^b^	Adjusted HR model 3 (95% confidence interval)^c^
Cohort 1	MI	**1.99 (1.85–2.15)**	**1.40 (1.30–1.50)**	**1.46 (1.33–1.60)**
	Stroke	**1.29 (1.18–1.41)**	1.00 (0.92–1.09)	0.92 (0.82–1.03)
	MI or stroke	**1.65 (1.56–1.75)**	**1.20 (1.14–1.27)**	**1.20 (1.12–1.29)**
Cohort 2	MI	**1.99 (1.83–2.15)**	**1.42 (1.32–1.54)**	**1.49 (1.35–1.65)**
	Stroke	**1.24 (1.12–1.36)**	0.99 (0.91–1.08)	0.92(0.82–1.04)
	MI or stroke	**1.63 (1.53–1.74)**	**1.21 (1.15–1.29)**	**1.23 (1.14–1.33)**
Cohort 3	MI	**1.42 (1.10–1.83)**	0.92 (0.73–1.16)	1.03 (0.75–1.42)
	Stroke	1.28 (0.95–1.72)	0.87 (0.66–1.15)	0.85 (0.58–1.24)
	MI or stroke	**1.33 (1.09–1.61)**	0.87 (0.73–1.04)	0.89 (0.69–1.15)

*SSDI* Social Security Disability Insurance

^a^Adjusted for age and sex

^b^Adjusted for variable included in model 1 and other CVD risk, and obesity. Other CVD risk was defined as baseline CCS 101 (coronary atherosclerosis and other heart disease), CCS108 (congestive heart failure; non-hypertensive), CCS127 (chronic obstructive pulmonary disease and bronchiectasis), CCS206 (spondylosis; intervertebral disc disorders; other back problems), CCS244 (other injuries and conditions due to external causes), CCS257 (other aftercare), CCS4 (mycoses), CCS49/50 (diabetes mellitus with or without complication), CCS55 (fluid and electrolyte disorders), CCS663 (screening and history of mental health and substance abuse codes), CCS98/99 (essential hypertension or hypertension with complications and secondary hypertension), number of physician visit in baseline, smoking, IP hospitalized during baseline (1/0), and use other anti-diabetes drugs during baseline

^c^Adjusted for variable included in model 2 and dual eligible (Medicare and Medicaid beneficiary), subsidy, and state buy in

There was no significant difference between the SSDI and Marketscan beneficiaries for the stroke outcome. Regarding the composite outcome of MI or stroke, cohort 1 had a HR of 1.21 (1.15–1.29) (absolute difference of 1.28 more myocardial infarcts or stroke per 1000 person-years in the SSDI versus Marketscan). Cohort 2 had a HR of 1.23 (1.14–1.33) (absolute difference of 1.40 more myocardial infarcts or stroke per 1000 person-years in the SSDI versus Marketscan). Once the population was conditioned to drug initiation, there was no difference for any of the CVD outcomes between SSDI beneficiaries and Marketscan (Table 2).

## Discussion

The rate of CVD events varied somewhat differently between cohorts of middle-aged patients with RA with SSDI benefits who are considered disabled to similar age patients RA who are enrolled in Marketscan, the majority of which are likely employed. Our Cohort 3, which anchored the start of follow-up at the time of initiation of first-time initiation or switch of bDMARD or tsDMARDs, used treatment change to homogenize RA disease activity. After anchoring on new treatment initiation and controlling for comorbidities, the CVD risk differences between patients with RA younger than 65 years of age commercially insured (considered to be employed) vs. SSDI beneficiaries disappeared. This suggests that the difference in CVD risk between SSDI beneficiaries and working individuals with RA were partially attributable to differences in the distribution of CVD risk factors and RA disease activity, proxied by treatment change for initiation or switching targeted therapies.

In the USA, 26% of adults live with a disability [1]. Within the causes of disability, diseases of the musculoskeletal system and connective tissue were the primary reason for disabled workers. Several studies have shown both an increased morbidity and mortality in disabled individuals when compared with non-disabled controls [11–13]. A recent US-based study showed that individuals with disability had a 51% increased risk of death (HR 1.51 95% CI 1.45–1.57) compared with non-disabled individuals, and the leading cause of death, different from that in non-disabled individuals, was cardiovascular disease [12]. The middle-aged individuals with RA that we analyzed in this study have a staggering number of comorbidities that could increase their risk for developing new conditions later in life, which could explain the differences observed in our study. It is worth highlighting that the absolute difference in MI only and the MI or stroke composite outcomes was small (i.e. < 2 events per 1000 patient-years) between the SSDI and Marketscan, even when there were significant differences observed in adjusted hazard ratios.

We made efforts to account for disease activity in our study by using initiation of bDMARD or tsDMARD and to also determine the effect of b/tsDMARD in lowering CVD risk as formal disease activity measures were not available in these claims databases. The results of our study were consistent with previous data about this topic where there were no differences in the risk for CVD after we limited the study population (cohort 3) to those who initiated a new b/tsDMARD or switched to a new b/tsDMARD [14]. There is a likelihood that some patients with high disease activity remained only on csDMARD, but our results suggest that they likely initiated or switched b/tsDMARD. This is because we observed that the incidence rate difference for MI or stroke between SSDI and Marketscan decreased between SSDI and Marketscan male patients with RA in the age group of 45–54. One might speculate on the reason for this observation, perhaps that this subgroup of men treated with RA therapies (cohort 3) has the most potential cardiovascular benefit of modern RA therapies to “equalize” their risk compared with those that are considered non-disabled/employed (Marketscan enrollment). Indeed, we see a similar drop in direction, albeit smaller in magnitude, in women. And perhaps men in their late 50 s and early 60 s then have age-related comorbidities overcome this effect, and the population-attributable risk related to RA disease control has less influence. However, these explanations are speculative on our part.

This investigation is among the first to examine the risk of CVD events among beneficiaries of the SSDI and individuals disabled with RA, compared with similarly aged working patients with RA. Strengths of this study included the large sample size, which increased our power to draw conclusions, and the use of SSDI data, which ensured that the patients in this study were indeed receiving CMS benefits because of disability. This was a longitudinal analysis, which is well suited to examine temporal associations between exposure and outcome. We considered in our population those beneficiaries that were dual eligible (Medicare and Medicaid) to avoid heterogeneity in medical and pharmacy coverage (because Medicaid pharmacy coverage is relatively uniform), which would have limited comparisons between groups. Our study was able to also examine the effect of bDMARD and tsDMARD, in lowering the risk for CVD among SSDI beneficiaries to that of those who remained working (Marketscan).

Limitations of our study include a lack of data regarding the reason for disability. While we assumed that disability was probably granted because the patient had RA, we believe this assumption is not particularly important, as RA is a disease that requires a high level of care and treatment. Regardless of the reason that SSDI benefits were granted, these patients had RA and were receiving DMARDs. Another limitation of our study was that we only enrolled individuals who obtained SSDI benefits and thus represent only those who were willing to endure an extensive judiciary process to obtain disability benefits. Therefore, our analyses may not have captured patients who had significant RA-related functional limitations but did not pursue disability benefits. Lastly, we were unable to examine the effect of social determinants of health, which are also well-known factors for CVD disease, given that Marketscan data is deidentified.

## Conclusion

Middle-aged patients with RA and beneficiaries of the SSDI had higher rates for CVD events compared with middle-aged individuals with RA who were privately insured (likely to be currently employed). These differences in CV event rates were partially explained by traditional CVD risk factors including comorbidities.

## Supplementary Information

**Additional file 1.** List of Disease Modifying anti-Rheumatic Drugs (DMARDs) Used for Cohorts 2 and 3.

**Additional file 2.** ICD-9/10-CM code for cohort, exclusion and outcome.

## Data Availability

Data for the study design can be found in the manuscript figures, tables, and supplementary materials. For further information of study design, please email the corresponding author, Jeffrey R. Curtis, MD MS MPH, at jrcurtis@uabmc.edu.

## Abbreviations

AHRQ: Agency for Healthcare Research and Quality
b: Biologic
bDMARD: Biologic disease-modifying antirheumatic drug
CI: Confidence intervals
CM: Clinical modification
CMS: Centers for Medicare & Medicaid Services
cs: Conventional synthetic
csDMARD: Conventional synthetic disease-modifying antirheumatic drugs
CVD: Cardiovascular disease
DMARDS: Disease-modifying antirheumatic drugs
HCPCS: Health Common Procedure Coding System
HIV: human immunodeficiency virus
HR: Hazard ratio
ICD: International Classification of Diseases
IR: Incidence rates
MI: Myocardial infarction
NDC: National Drug Code
RA: Rheumatoid arthritis
SD: Standard deviation
SMDs: Standardized mean differences
SSDI: Social Security Disability Insurance
ts: Targeted synthetic
tsDMARD: Targeted synthetic disease-modifying antirheumatic drug
