# Supplementary material for: Comparing cardiovascular risk of patients with rheumatoid arthritis within the Social Security Disability Insurance with those commercially insured
Source: Arthritis Res Ther. 2022 Aug 22;24:202. doi: 10.1186/s13075-022-02847-1 (PMC9396772; doi:10.1186/s13075-022-02847-1)
Supplement: Supplementary file 1 — Additional file 1. List of Disease Modifying anti-Rheumatic Drugs (DMARDs) Used for Cohorts 2 and 3. [file 13075_2022_2847_MOESM1_ESM.docx]

**Supplement Table 1:** List of Disease Modifying anti-Rheumatic Drugs (DMARDs) Used for Cohorts 2 and 3

| **csDMARD** | **bDMARD** | **tsDMARD** |
| --- | --- | --- |
| Methotrexate | Adalimumab | Tofacitinib |
| Sulfasalazine | Etanercept |  |
| Hydroxychloroquine | Infliximab |  |
| Leflunomide | Certolizumab |  |
|  | Golimumab |  |
|  | Sarilumab |  |
|  | Tocilizumab |  |
|  | Abatacept |  |
|  | Rituximab |  |
