# Supplementary material for: Comparing cardiovascular risk of patients with rheumatoid arthritis within the Social Security Disability Insurance with those commercially insured
Source: Arthritis Res Ther. 2022 Aug 22;24:202. doi: 10.1186/s13075-022-02847-1 (PMC9396772; doi:10.1186/s13075-022-02847-1)
Supplement: Supplementary file 2 — Additional file 2. ICD-9/10-CM code for cohort, exclusion and outcome. [file 13075_2022_2847_MOESM2_ESM.docx]

Supplementary table 2: ICD-9/10-CM code for cohort, exclusion and outcome

|  |  | ICD-9-CM diagnosis code | ICD-10-CM diagnosis code |
| --- | --- | --- | --- |
| Cohort | Rheumatoid arthritis | 714.0x, 714.2x, 714.81 | M05.*, M06.* excluding M06.1, M06.4. |
| Exclusion | Ankylosing spondylitis | 720.0x | M45.0-M45.9 |
|  | Inflammatory bowel disease |  |  |
|  | Psoriatic arthritis | 696.0x | L40.51-L40.54, L40.59 |
|  | Psoriasis | 696.1x | L40.0-L40.4, L40.8 |
|  | Malignancy | 140 -172.xx, 174 -209.xx,  V10.xx | C00-C43.*, C45-C99.*, Z85.* |
|  | HIV | 042-044.xx, V08.xx | B20.* |
|  | Myocardial infarction | 410.xx, 412.xx | I21.*, I22.* |
|  | Stroke | 430.xx, 431.xx, 433.x1, 434.x1, 436.xx | I60.*, I61.*, I63.*, I67.89 |
| Outcome | Myocardial infarction | 410.x1 | I21.* |
|  | Stroke | 430.xx, 431.xx, 433.x1, 434.x1, 436.xx | I60.*, I61.*, I63.*, I67.89 |

X: any 1one digits of number or missing

*: Any number of digits, alphabetic, or missing.
